# Copper Deficiency-Induced Neuropathy After Bariatric Surgery Disguised as Demyelinating Disease: A Case Report

**DOI:** 10.7759/cureus.22705

**Published:** 2022-02-28

**Authors:** Adina Amin, Neil C Khoury, Miguel Lacayo, Sofya Kostanyan

**Affiliations:** 1 Internal Medicine, NewYork-Presbyterian Brooklyn Methodist Hospital, Brooklyn, USA

**Keywords:** demyelinating disease, acquired peripheral neuropathy, neuropathy, copper deficiency, bariatric surgery complications

## Abstract

Neuropathy may arise from many different etiologies - from diabetes and nerve compression to viral infections and chemotherapy side effects; many patients suffer from neuropathic symptoms. While some etiologies produce irreversible neuropathy, others, such as vitamin and mineral deficiencies, lead to a possibly reversible disease process once treated. General clinicians should strive for early and prompt diagnosis of copper deficiency neuropathy whenever possible, especially in patients with normal vitamin B12 levels who present with a subacute gait disorder or prominent sensory ataxia. We present a case of a 73-year-old female with a surgical history of Roux-en-Y gastric bypass (RYGB) 20 years prior, who presented with difficulty with ambulation due to sensory ataxia and bilateral, ascending, sensory neuropathy, who was diagnosed with acquired copper deficiency-induced myeloneuropathy.

## Introduction

Bariatric surgery has arisen as a tremendously helpful modality for improving weight loss and other comorbidities, such as type 2 diabetes [1]. The number of bariatric and metabolic surgeries has been on the rise, up to 216,000 procedures in 2016 (a marked 10% increase from 2015) [2].

Despite the many proven advantages of bariatric surgery, various postsurgical complications may occur. Some complications include gallstone formation due to rapid weight loss (up to 38% of patients), diarrhea with a sympathetic nervous system response due to dumping syndrome (up to 50% of patients), and small bowel obstruction (3%-5% of patients) [3]. Bariatric surgery may also lead to neurological complications occasionally due to mechanical or inflammatory mechanisms but more often due to vitamin deficiencies [4]. Patients may develop deficiencies of specific vitamins and minerals that are absorbed throughout the gastrointestinal tract. These include but are not limited to cobalamin, folate, thiamine, copper, vitamin D, and other fat-soluble vitamins [5].

Of the mineral deficiencies, copper deficiency presents in 9.6%-18.8% of post-Roux-en-Y gastric bypass (RYGB) patients. This can be particularly troublesome if not appropriately diagnosed and treated due to its hematologic and neurological manifestations [6]. Copper distribution is mediated by binders such as ceruloplasmin and albumin. About 30%-40% of dietary copper is typically absorbed throughout the gastrointestinal tract [7]. Most copper absorption occurs in the small intestine, particularly in the duodenum; however, some copper can be absorbed in the stomach as well. After certain bariatric surgeries, such as RYGB, ingested food bypasses the duodenum, resulting in significantly reduced copper absorption.

Copper is essential for the process of erythropoiesis; therefore, copper deficiency may cause anemia, neutropenia, or pancytopenia [8]. Copper deficiency may lead to symptomatic anemia, presenting with weakness, shortness of breath, dizziness, paleness, or fatigue. In addition, the neurological effects of copper deficiency, such as gait difficulty (due to dorsal column dysfunction), lower limb spasticity, and neuropathy, could potentially mimic the presentation of demyelinating disease [8]. Although less reported, copper deficiency has been shown to cause cerebral demyelination, which may further lead to diagnostic mimicking and potential misdiagnosis [8].

## Case presentation

A 73-year-old female with a past medical history of cobalamin deficiency, postsurgical hypothyroidism, anxiety, and hypertension and surgical history of Roux-en-Y gastric bypass 20 years prior presented to the emergency department for difficulty ambulating due to painful neuropathy. She had severe “pins and needles” in her feet, worsening for the past three months. The sensation had progressed to involve her mid-shins and her bilateral fingertips. She stated that she often bumped into things and had fallen twice last week due to an inability to feel where she was stepping. She was informed that her cobalamin levels were low on initial investigation; however, her symptoms did not improve with cobalamin supplementation. Her primary care physician had prescribed pregabalin and gabapentin, which improved her symptoms slightly. She also had recently completed outpatient nerve conduction studies (NCS) and electromyography (EMG), which supposedly suggested that the patient had a demyelinating peripheral neuropathy. She denied fevers, chills, recent illness, or diarrhea. Her vital signs on admission were within normal limits. On examination, she was pleasant and well-appearing. She had multiple bruises, cuts, and wounds on her left lower extremity, decreased proprioception bilaterally, and decreased sensation to light touch to her mid-shins bilaterally and symmetrically. Sensation was intact in her bilateral upper extremities, hands, and fingers. Admission laboratory results were significant for thyroid-stimulating hormone (TSH) of ​​56.600 μIU/mL, vitamin B12 ​​of 1414 pg/mL, methylmalonic acid of ​​140 nmol/L, iron of 19 ug/dL, iron saturation of 5.15%, ferritin of 43.1 ng/mL, and hemoglobin of 11.7 g/dL (Table 1). Cranial computed tomography (CT) showed mild cerebral volume loss and microvascular ischemic change, without any evidence of acute intracranial hemorrhage, mass, or infarct.

**Table 1:** Pertinent laboratory test values on admission with normal ranges for reference

Laboratory test	Test value	Normal range
Thyroid-stimulating hormone (TSH)	56.600 μIU/mL	0.360–0.3.740 IU/mL
Vitamin B12	1414 pg/mL	200–1100 pg/mL
Methylmalonic acid	140 nmol/L	87–318 nmol/L
Iron	19 ug/dL	65–175 ug/dL
Iron saturation	5.15 %	20%–55%
Ferritin	43.1 ng/mL	8–252 ng/mL
Hemoglobin	11.7 g/dl	11.2–15.7 g/dl
Platelets	271 × 10^3^/uL	163–369 × 10^3^/uL
White blood cells	8.34 × 10^3^/uL	3.98–10.04 × 10^3^/uL

She was admitted to the general medical floor and was discharged two days later to the acute rehabilitation floor, where further workup was completed. A lumbar puncture resulted in normal CSF studies, and she was given five days of intravenous immune globulin (IVIG) for the empiric treatment of possible chronic inflammatory demyelinating polyradiculoneuropathy (CIDP). When her copper level and ceruloplasmin resulted after several days of admission, they were both low (copper: 27 mcg/dL; ceruloplasmin: 14 mg/dL), consistent with copper deficiency. She was then started on intravenous copper supplementation, which resulted in slight improvement in her neurological symptoms. She was discharged on day 15 to complete a two-week course of oral copper supplementation. One month after discharge, her symptoms were reassessed via follow-up phone call. Her symptoms were still present; however, they did not significantly change or worsen, indicating overall slight improvement.

## Discussion

In addition to weight loss, post-bariatric surgery patients may experience a plethora of benefits, including but not limited to 72%-98% resolution of gastroesophageal reflux, 82% cardiovascular disease risk reduction, 90% improvement in hepatic steatosis, and 55% improvement or resolution of depression [9]. Despite these impressive benefits, physicians and patients should be aware of the potential risks that come along with these procedures. Of the aforementioned complications, vitamin and mineral deficiencies are fairly common and can be easily screened for and treated.

Copper deficiency, in particular, can lead to significant morbidity in the post-bariatric surgery patient population. Although the mechanism underlying neurological damage due to copper deficiency is unclear, the enzymes and pathways that require copper are well established. Copper plays a critical role in pathways such as electron transport chain and oxidative phosphorylation, and serotonin synthesis [8]. Other etiologies of copper deficiency include zinc excess (often secondary to parenteral zinc supplementation during chronic dialysis or denture wearers who use zinc-containing adhesives), iron supplementation, and impaired copper transport (Menkes syndrome) [8].

Our patient went through extensive workup, including a nerve conduction study, EMG, lumbar puncture, a five-day course of IVIG, and 15-day hospital admission, to diagnose a mineral deficiency that could have potentially been discovered on screening laboratory tests. In this case, the diagnosis was further delayed due to diagnostic anchoring on an outpatient diagnosis of a demyelinating disease such as CIDP and the fact that the serum copper and ceruloplasmin tests took several days to result. The ceruloplasmin level was sent on admission day three and took one day to result; however, the copper level was sent on day five of admission and took three days to result.

In 2016, the American Society for Metabolic and Bariatric Surgery updated their integrated health nutritional guidelines for the surgical weight loss patient to include a grade C recommendation for at least annual screening of copper status with serum copper and ceruloplasmin levels in post-RYGB patients, even in the absence of clinical signs or symptoms of deficiency [10]. This is even more important in post-biliopancreatic diversion/duodenal switch (BPD/DS) patients, in whom copper deficiency has been reported up to 90% [10].

The treatment of copper deficiency myelopathy has shown to lead to slight symptom improvement and prevention of worsening disease process [11]. Once the proper diagnosis of copper deficiency was established, our patient's distressing neuropathy slightly improved with intravenous copper supplementation once the proper diagnosis was made; however, most importantly, further neurological deterioration was likely prevented.

There is no gold standard diagnostic test for copper deficiency myelopathy. There are also no randomized control trials available to support guideline-directed dosing for prophylactic copper supplementation status post-bariatric surgery or for the treatment of diagnosed copper deficiency myelopathy, although suggested doses are available (Tables 2-3) [11]. Thus, period assessment of serum copper is integral to determine the efficacy of replacement.

**Table 2:** Suggestions for prophylactic copper supplementation dosing status post-bariatric surgery BPD/DS: biliopancreatic diversion/duodenal switch, RYGB: Roux-en-Y gastric bypass, LAGB: laparoscopic adjustable gastric band, SG: sleeve gastrectomy

Type of bariatric surgery	Copper supplementation dose
BPD/DS or RYGB	2 mg PO daily
LAGB or SG	1 mg PO daily

**Table 3:** Suggestions for copper deficiency myeloneuropathy treatment dosing

Severity of copper deficiency myeloneuropathy	Copper supplementation dose and frequency
Mild–moderate	3–8 mg PO until levels normalize
Severe	2–4 mg IV × six days

## Conclusions

Of the many different etiologies of neuropathy for a physician to consider, deficiencies in vitamins and minerals can be diagnosed cost-effectively with blood tests without the need for expensive or invasive investigations. Although copper deficiency is not completely reversible, physicians can halt the progression of bothersome neuropathic symptoms with timely treatment. Physical examination clues that would raise suspicion for this pathology include a positive Babinski sign, a positive Romberg sign, or decreased vibration, indicating dorsal column involvement. However, physicians should strive for early diagnosis in asymptomatic patients to prevent potentially irreversible neurological damage. Thus, early diagnosis of copper deficiency in post-bariatric surgical patients by yearly screening of copper and ceruloplasmin levels can prevent significant morbidity, as well as potentially unnecessary and invasive testing. Randomized controlled trials are needed to help determine standardized guidelines for the treatment of copper deficiency myelopathy.
